# Exploring the Relationship Between Nonnutritive Sweeteners and Nutrient Intake: Findings from the ELSA-Brasil Baseline Study

**DOI:** 10.3390/nu17111778

**Published:** 2025-05-24

**Authors:** Taiz Karla Brunetti Moreira, Fernanda Duarte Mendes, Hully Cantão dos Santos, Gabriela Callo Quinte, José Geraldo Mill, Maria del Carmen Bisi Molina, Carolina Perim de Faria

**Affiliations:** 1 Post-Graduation Program in Nutrition and Health, Federal University of Espírito Santo, Vitória 29040-090, Brazil; taiz_karla@hotmail.com (T.K.B.M.); fernanda.mendes@edu.ufes.br (F.D.M.); mdcarmen2007@gmail.com (M.d.C.B.M.); 2 Post-Graduation Program in Public Health, Federal University of Espírito Santo, Vitória 29040-090, Brazil; hully.santos@edu.ufes.br (H.C.d.S.); gavizon7@hotmail.com (G.C.Q.); josegmill@gmail.com (J.G.M.)

**Keywords:** sweetening agents, nonnutritive sweeteners, diet, nutrient intake, carbohydrates

## Abstract

Background/Objectives: This study examines the association between the regular consumption of nonnutritive sweeteners (NNSs) and dietary intake among non-diabetic participants from the Brazilian Longitudinal Study of Adult Health (ELSA-Brasil). Methods: The sample included 9226 individuals aged 35–74 years, with data collected during ELSA-Brasil’s baseline. Regular NNS consumption, defined as using NNS-sweetened products at least once daily. Results: regular NNS use was prevalent in 25.7% of the participants, with higher usage among women, older age groups, higher BMI categories, higher education, and income levels. Multivariate analysis adjusted for sociodemographic and lifestyle factors indicated that NNS use was significantly associated with reduced daily energy (−74.29 kcal), total carbohydrate (−23.68 g), and simple carbohydrate (−11.24 g) intake, while positively associated with increased protein (7.38 g) consumption. Conclusions: In conclusion, these findings indicate that while there may be some carbohydrate/protein compensation, regular use of nonnutritive sweeteners (NNSs) is associated with fewer calories and carbohydrates, particularly sugars. This suggests that NNSs could be a useful tool for reducing overall caloric and sugar intake in the diet.

## 1. Introduction

Products with reduced sugar content have been used as a viable alternative for preventing diseases related to the high consumption of sugars by the population and for cases in which metabolic alterations demand the replacement/reduction in the consumption of this nutrient [1].

Dietary products containing nonnutritive sweeteners (NNSs) have been ingested not only by individuals with Diabetes Mellitus (DM) and other pathologies but also by subjects aiming for body weight control reduction [2,3].The reported effects of these additives on energy and macronutrient intake vary widely, ranging from the intended outcomes like reduced energy and carbohydrate intake to opposite, counterintuitive results.

Some studies have shown that NNSs can help lower calorie intake by substituting for sugar, leading to modest reductions in overall energy consumption and carbohydrate intake, as seen in research by Rogers and Appleton (2021) [4]. However, other studies have reported that NNS consumption may increase feelings of hunger and subjective appetite, leading to higher overall food intake. For instance, Rogers and Blundell (1989) [5] observed that sweetness without caloric content, such as in the case of saccharin, can stimulate hunger. Similarly, Rolls et al. (1989) [6] noted that consumption of low-calorie foods, including those with NNSs, can lead to increased food intake in subsequent meals. This compensatory behavior often involves an increased intake of fats and proteins, as highlighted by Sylvetsky and Rother (2018) [7], potentially undermining the caloric deficit intended by the use of NNSs. Additionally, some studies, such as Swithers (2013) [8], suggest that NNSs may contribute to metabolic derangements, further complicating their role in weight management. Overall, while NNSs can help reduce caloric intake under certain conditions, their effects on appetite regulation and food consumption can lead to outcomes opposite to those intended, including weight gain and altered macronutrient balance.

Given the rising consumption of nonnutritive sweeteners (NNSs) among the general population over recent decades, the growing concern about their potential effects and the lack of robust studies highlight the need to investigate how regular consumption of NNSs associates with energy and macronutrient intake, particularly in populations without evident glycemic alterations. Conducting such research is critical for understanding how NNSs impact metabolic health in diverse contexts and informing public health strategies in a country where NNS use is becoming more widespread.

## 2. Materials and Methods

The study sample used for this analysis came from the Brazilian Longitudinal Study of Adult Health (ELSA-Brasil), a cohort study that evaluated, in its first wave, between August 2008 and December 2010, 15,105 women and men aged 35 to 74 who were active or retired civil servants. They were recruited from five higher education and one research institution in Salvador, Vitória, Belo Horizonte, Rio de Janeiro, São Paulo, and Porto Alegre [9].

Our initial sample (N = 15,105) included all adult individuals from the first wave. We excluded from the analysis those who did not respond to the food intake questionnaire, those who underwent bariatric surgery before data collection, those who changed their diets in the previous six months (potentially biasing regular food intake results), and those with implausible energy consumption (<300 or >6500 kcal) [10]. Additionally, we excluded individuals with a laboratory diagnosis, a report of a previous diagnosis, or those using medication for Diabetes Mellitus, as diabetics often have dietary restrictions and medical interventions that could alter eating habits and metabolic responses. These unique dietary patterns could affect how nonnutritive sweeteners (NNSs) influence their overall diet, making the study’s results less generalizable to non-diabetic individuals.

To assess food consumption, a Food Frequency Questionnaire (FFQ) was used; it contains 114 items and 14 checking questions, its objective was to assess participants’ habitual consumption over the last 12 months. The elaboration and validation of the FFQ are described in detail in the articles by Molina et al. 2013a and 2013b [11,12].

The nutritional composition of food items was estimated from the Nutrition Data System for Research (NDSR)—University of Minnesota. This international food composition table was used to analyze a more significant number of nutrients and substances [11,12]. For the analysis presented in this work, food consumption data were adjusted for energy consumption using the method proposed by Willett et al. (1998) [13]. Willett’s method for adjusting nutrient intake by energy intake is designed to control for the confounding effect of total calorie consumption when analyzing the relationship between macronutrient consumption and health outcomes. It is performed by calculating the nutrient intake per 1000 kilocalories of total energy intake, thereby standardizing nutrient consumption relative to total caloric intake to account for differences in overall energy consumption among individuals. Since individuals with higher energy intake tend to consume more macronutrients overall, adjusting for energy intake helps isolate the effects of specific macronutrients independent of total calorie consumption [13].

The following nutrients were selected: energy (kcal/day), fat (g/day), total carbohydrate (g/day), simple carbohydrate (g/day), complex carbohydrate (g/day), protein (g/day), animal protein (g/day), vegetable protein (g/day), total fiber (g/day), soluble fiber (g/day), and insoluble fiber (g/day).

Regular consumption of nonnutritive sweeteners (NNSs) was considered when one of the following products sweetened with NNSs was used at least once daily: soda, coffee, natural juice, industrialized juice, artificial juice, tea, or mate tea.

Anthropometric variables of weight and height were collected by trained personnel according to established techniques [14]. The height of the participants was measured with a wall stadiometer (Seca^®^, Hamburg, Germany, Model 216) affixed to a smooth wall without a baseboard; the participant was wearing traditional study clothing, barefoot, in a standing position, with head, shoulders, buttocks, and heels touching the wall, looking straight ahead/Frankfort plane. While still barefoot, with the head straight, arms along the body, and looking forward, body weight was measured using an electronic scale (Toledo^®^, São Bernardo do Campo, São Paulo, Brazil, model 2096PP) with a capacity of 200 kg and accuracy of 50 g.

BMI was calculated by dividing weight (kg) by squared height (m^2^) and classified according to the cutoff points recommended by the WHO [15]: thinness < 18.5 kg/m^2^; eutrophy 18.5–24.9 kg/m^2^; overweight 25–29.9 kg/m^2^; obesity > 30 kg/m^2^.

Sociodemographic characteristics were collected through a questionnaire and include gender; age, divided into age groups: 35 to44, 45 to 54, 55 to 64, 65 to 74 years; schooling, classified as elementary, secondary, and higher, according to the highest educational level achieved; per capita income, based on the total net income of all family members in the last three months, divided by the number of people dependent on this income, categorized according to the minimum wage in force at the time of analysis—BRL 937.00; and self-reported race/skin color (white, black, yellow, brown and Indigenous) according to the categories of the Brazilian Institute of Geography and Statistics [16].

Still, regarding lifestyle, the study variables include information about the practice of physical activity during leisure time, measured using the International Physical Activity Questionnaire (IPAQ) extended version, validated in Brazil [17], and classified as weak, moderate, and strong.

Alcohol consumption among participants was collected using a structured Alcohol Consumption Questionnaire based on the National Center for Health Statistics Questionnaire (1994) [18]. Alcohol consumption was categorized among participants who reported consuming or not consuming alcoholic beverages at the time of data collection.

To classify the participant in terms of smoking, they should answer the following questions: Are you or have you ever been a smoker, that is, have you smoked at least 100 cigarettes (five packs of cigarettes) throughout your life?” and “Do you currently smoke cigarettes?” The three categories considered were: “never smoked”, “ex-smoker” and “current smoker”.

Study variables were described using measures of central tendency (mean) and measures of dispersion (standard deviation—SD) for continuous variables and percentages for categorical variables. Student’s *t*-test and ANOVA were used, followed by Tukey’s test for the analysis of differences in means and the chi-square test for the analysis of differences in proportions.

The normality of continuous variables was not tested due to the large sample size. In such cases, parametric methods can be used regardless of the sample distribution, because large samples tend to approximate a normal distribution [19].

Initially, simple associations between food consumption variables (outcome) and regular consumption of NNSs (exposure) were tested, resulting in the identification of nutrients to be examined in the multivariate models. Then, sociodemographic and lifestyle variables were considered for their association with exposures and outcomes, with those having a *p*-value < 0.05 in this analysis included in the adjusted models.

Multivariate analysis was performed to model the relationship between the multiple exposures and kcal and macronutrient intake accounting for potential correlations among them. This was based on crude linear regression models (association between food consumption and NNS use) that were then adjusted for previously tested sociodemographic and lifestyle variables. Exploratory stratified analyses were also carried out, first by sex and BMI category separately and later by both variables together, seeking to identify different behaviors in any of these proposed situations to assess the need to perform stratified multivariate analyses.

The significance level for all tests was set at α = 0.05. Statistical procedures were performed using SPSS for Windows, version 23, and Stata 18.0.

## 3. Results

A total of 5879 participants were excluded according to pre-established exclusion criteria (28 lacking information on the diet; 124 bariatric patients, 4599 who reported changing diet over the previous 6 months, 304 had implausible diets, and 824 were diabetics). Thus, 9226 eligible individuals were included in the study, whose general characteristics are described in Table 1.

The prevalence of regular consumption of NNSs in the studied sample was 25.7% (95% CI 24.8–26.6). The average energy consumption was 2891.4 ± 1011.5 kcal/day, 86.6 ± 15.9 g/day of fat, 360.8 ± 56.3 g/day of carbohydrates, and 125.9 ± 25.2 g/day of protein.

Analyzing the association between the exposure variable (regular consumption of NNSs) and sociodemographic and lifestyle variables, it was observed that women were the biggest consumers of NNSs, with a prevalence of 30.5%. The use of these sweeteners tends to increase with age, as well as with BMI categories (Table 1).

It is also observed that the highest prevalence of use of these additives is found in the highest education and per capita income categories. The following were also favorable to the consumption of NNSs: not being married, having diabetes run in the family, having high blood pressure, and alcohol consumption. Tobacco use was not associated with NNS consumption (Table 1).

Table 2 and Table 3 show the analysis of the relationships between sociodemographic and lifestyle variables and the outcome variables (energy and nutrient intake); it can be seen that only the family history of diabetes mellitus was not related to any of the studied variables.

Regarding the energy and macronutrient consumption of the participants, Table 4 summarizes the average daily intake of energy and macronutrients of the total sample and the subgroups of non-users and users of NNSs. The mean difference between users and non-users (ΔΔ) and the *p*-value for statistical significance are also reported. Those who use NNSs consumed lower amounts of energy, carbohydrates (total, simple, and complex), and vegetable protein, but higher amounts of fat, protein (total and animal), and soluble fiber. Regarding total and insoluble fibers, there was no statistically significant difference between regular and non-consumers of NNSs (Table 4).

The results obtained from the stratified analyses demonstrate outcomes similar to the those of the general analyses, indicating that the variables sex and BMI can be used as an adjustment in multivariate models, without requiring differentiated analyses.

After identifying the bivariate associations between exposure, outcome, and adjustment variables, crude and adjusted multivariate linear regression models were used. The results of these models are presented in Table 5. The crude analysis shows that those who use NNSs consumed lower amounts of energy, carbohydrates (total, simple, and complex), vegetable protein, and soluble fiber, but higher amounts of protein (total and animal). In the adjusted model, it is observed that the consumption of energy, total and simple carbohydrates, and total and animal proteins remained associated with the regular use of NNSs (the non-consumption of NNSs serves as the reference category). These associations were positive for total protein and its animal fraction, while for energy and total and simple carbohydrates, the association was negative.

## 4. Discussion

The present study showed a significant association between the use of nonnutritive sweeteners (NNSs) and higher consumption of proteins (total and animal), as well as reduced consumption of total and simple carbohydrates and total calories. No association was found between the use of nonnutritive sweeteners (NNSs) and the intake of total fats, fiber (soluble and insoluble fiber), vegetable protein, and complex carbohydrates. These findings align with previous studies. For instance, the “SU.VI.MAX” cohort study on a national sample of French adults (n = 4278) reported similar trends, where increased NNS use was associated with higher protein and lower carbohydrate consumption [20]. Another study in the UK found higher fat and protein consumption among individuals following a sugar-reduced diet compared to those on a regular diet [21].

The lack of association with complex carbohydrates can be explained by the primary use of nonnutritive sweeteners (NNSs) to replace simple carbohydrates, which provide sweetness in foods and beverages without changing the composition of complex carbohydrates in diets. Additionally, the concept of the sugar-fat seesaw presented by McColl in 1988 [22] and later described by Benton et al. (2005) [23] as a “sugar-fat balance”, describes that diets high in fat tend to be low in sugar, and vice versa. When NNSs replace sugar, there may be a compensatory increase in fat and protein intake to balance the reduced carbohydrate energy [24].

The mechanism of macronutrient substitution suggests that removing calories from sugar can lead to increased consumption of other macronutrients to restore energy balance [24]. Rosado and Monteiro (2001) [25] also noted that replacing sugar with NNSs might promote higher lipid intake. This compensatory mechanism is further supported by Richter and Lünse (2007) [26] who observed that replacing sugar with NNSs often requires adding fat to maintain desirable food texture and taste.

Anderson et al. (2012) [27] reviewed data from the NHANES 2001–2002, confirming that NNS consumers have higher fat and protein intake and lower carbohydrate and added sugar intake compared to non-consumers, aligning with the direction of the present study’s findings. However, while caloric compensation occurs, it does not seem to surpass the reductions in CHO to cause an annulment of the inverse association with total daily energy intake.

On similar grounds, the American Dietetic Association and the American Diabetes Association also advocate for NNSs as a tool to lower energy consumption, emphasizing that even with some energy compensation, NNSs can help maintain a negative energy balance [28,29,30]. Moreover, the effectiveness of NNSs in reducing total caloric intake is enhanced when integrated into broader dietary and lifestyle changes [7,31].

Likewise, a somewhat recent systematic review and meta-analysis by Rogers and Appleton (2021) [4] focusing on intervention studies showed that NNSs seem to be a viable alternative for weight management through the amount of simple carbohydrates they can displace in the diet. This finding is further supported by the systematic review published by Nathur and Bakshi in 2004 [32]. Furthermore, the 2015 Dietary Guidelines Advisory Committee (DGAC) scientific report supports the idea that replacing sugar with low-calorie sweeteners can reduce calorie intake, body weight, and adiposity in the short term [33]. Nonetheless, the report advises caution due to insufficient long-term evidence.

The contrasting results between our study and findings from the Cancer Prevention Study-3, highlight the importance of considering cultural and dietary context when interpreting associations between nonnutritive sweetener (NNS) use and diets. While the U.S. study reported lower overall diet quality among higher NNS consumers, our analysis of the ELSA-Brasil cohort found that regular NNS use was associated with reduced intake of total and simple carbohydrates and increased protein consumption. These differences may reflect distinct patterns of NNS use across populations. In Brazil, NNS use appears to be more common among individuals with greater health awareness, higher education, and income—groups that may be actively pursuing dietary improvements. Conversely, in the U.S., NNS consumption may be more closely tied to processed or diet-labeled products that do not necessarily align with overall healthy eating patterns. These contextual factors suggest that the role of NNS in shaping diet quality is not uniform across countries and should be evaluated in light of regional food cultures, dietary norms, and consumer motivations [34].

Also, as non-sugar sweeteners (NNSs) may serve as a strategy to reduce sugar intake, and while their cost could be discussed as a potential limitation, this concern appears less relevant in our context, given that our sample’s income is significantly higher than the minimum wage observed in Brazil. Nevertheless, each country has its own financial environment and consumer purchasing power, and the feasibility of this strategy should be assessed individually according to local economic conditions.

Although these results suggest an association between NNSs and lower carbohydrate intake, it is crucial to consider the broader context of NNS use. The World Health Organization (WHO) recently advised against the use of non-sugar sweeteners for weight control, citing potential long-term adverse effects and limited evidence of sustained benefits [35].

The strengths of this study count on the ELSA-Brasil database, which is known for the quality of the collected data. We also carefully tried to determine the regular use of NNSs and not only episodic or intermittent use, while focusing on addressing potential confounding variables through the careful analysis performed. In addition, we used multivariate multiple linear regression, a robust technique that can detect patterns that might be missed when analyzing each outcome separately. It is important to acknowledge that the cross-sectional design of this study limits the ability to infer causality. Consequently, the observed associations may be partially explained by reverse causality, whereby individuals who are already engaged in dietary modifications—such as reducing simple sugar intake or limiting animal protein consumption—may be more likely to use NNS-containing products, reflecting pre-existing dietary preferences rather than effects attributable to NNS consumption itself. Also posing a limitation, it is important to acknowledge the use of a validated semi-quantitative Food Frequency Questionnaire, which, despite all efforts, may be subject to memory bias. Additionally, although our sample is large and comprehensive, it cannot be considered representative of the Brazilian population. 

## 5. Conclusions

The main objectives related to the use of NNSs are to promote glycemic control in individuals with diabetes and to reduce energy consumption aimed at weight loss in individuals who are overweight and obese [36]. Our findings show that the regular use of NNSs is associated with higher consumption of proteins and lower total energy and carbohydrate intake; this being independent of nutritional status, sociodemographic, and health-related characteristics.

In conclusion, the use of NNSs seems to demonstrate potential benefits; however, it is essential to use NNSs cautiously due to the uncertainty of long-term effects and possible compensatory behaviors. Future research should continue to explore the long-term effects of NNSs on dietary patterns, nutritional status, and overall health.

## Figures and Tables

**Table 1:** Sample description and prevalence of regular use of NNSs among non-diabetic participants of ELSA-Brazil according to demographic, socioeconomic, and health characteristics (2008–2010).

Characteristics	Sample		Regular Use of NNS ^1^	
	N	%	Prevalence	95% CI
**Gender**				
Male	4326	46.9	20.3	19.1–21.5
Female	4900	53.1	30.5	29.2–31.8
**Ethnicity**				
Black	1292	14.2	18.5	16.5–20.7
Brown	2522	27.7	22.0	20.4–23.7
White	4996	54.8	29.4	28.2–30.7
Yellow/Indigenous	305	3.3	25.6	21.0–30.8
**Age (years)**				
35–44	2047	22.2	20.4	18.7–22.2
45–54	3661	39.7	24.4	23.1–25.8
55–64	2571	27.9	29.5	27.8–31.3
65–74	947	10.3	32.0	29.1–35.0
**Marital status**				
Married	6229	67.5	25.0	23.9–26.1
Unmarried	2997	32.5	27.3	25.7–28.9
**BMI classification**				
Thinness	115	1.2	8.7	4.8–15.3
Eutrophy	3817	41.4	20.1	18.9–21.4
Overweight	3612	39.2	28.4	27.0–29.9
Obesity	1677	18.2	34.0	31.8–36.3
**Family history of Diabetes**				
No	5868	64.5	24.8	23.7–25.9
Yes	3224	35.5	27.9	26.3–29.4
**Hypertension**				
No	6295	68.3	24.6	23.6–25.7
Yes	2926	31.7	28.1	26.5–29.7
**Education level**				
Middle School	893	9.7	15.1	12.9–17.6
High School	2737	29.7	18.9	17.5–20.4
University	5596	60.7	30.8	29.6–32.0
**Per Capita Income**				
Up to 1 MW ^2^	2977	32.4	16.2	14.9–17.6
From 1 to 2 MW	2915	31.7	26.3	24.7–27.9
More than 2 MW	3300	35.9	33.8	32.2–35.5
**Alcohol Consumption**				
No	1631	19.7	20.8	18.9–22.9
Yes	6662	80.3	26.8	25.8–27.9
**Tobacco Consumption**				
No	5269	57.1	26.2	25.0–27.4
Yes	3957	42.9	25.1	23.8–26.5
**Leisure Physical Activity**				
Weak	7122	78.3	24.4	23.4–25.4
Moderate	1199	13.2	31.3	28.7–34.0
Strong	780	8.6	30.5	27.4–33.8

^1^ NNS: Nonnutritive Sweeteners, ^2^ MW: Minimal Wage.

**Table 2:** Association between sociodemographic and lifestyle variables and nutrient intake (kcal, fats, carbohydrates (simple and complex)). ELSA-Brazil. 2008–2010 ^1^.

	Kcal	Fat (g)	CHO (g)	CHO Simple (g)	CHO Complex (g)
	Mean ± SD	Mean ± SD	Mean ± SD	Mean ± SD	Mean ± SD
**Gender**					
Male	3181 ^a^ ± 1028	86.3 ± 15.5	357.0 ^a^ ± 55.6	130.7 ^a^ ± 42.8	143.4 ^a^ ± 39.5
Female	2636 ^b^ ± 925	86.8 ± 16.2	364.2 ^b^ ± 56.6	153.5 ^b^ ± 45.5	132.7 ^b^ ± 38.6
**Ethnicity**					
Black	3181 ^a^ ± 1094	82.3 ^a^ ± 15.6	373.4 ^a^ ± 56.5	146.8 ^a^ ± 50.5	140.9 ^a^ ± 39.9
Brown	3030 ^b^ ± 1048	84.4 ^b^ ± 15.7	366.8 ^b^ ± 56.2	142.2 ^b^ ± 47.8	141.9 ^ac^ ± 40.2
White	2755 ^c^ ± 948	88.9 ^c^ ± 15.7	353.8 ^c^ ± 55.2	141.9 ^b^ ± 43.0	134.4 ^b^ ± 38.4
Yellow/Indigenous	2766 ^cd^ ± 960	84.7 ^ab^ ± 15.3	372.9 ^ab^ ± 56.3	141.7 ^ab^ ± 46.5	147.3 ^c^ ± 40.1
**Age (years)**					
35–44	2999 ^a^ ± 1022	89.0 ^a^ ± 15.0	356.6 ^a^ ± 52.9	138.7 ^a^ ± 42.9	142.7 ^a^ ± 38.7
45–54	2933 ^a^ ± 1010	87.2 ^b^ ± 15.6	358.5 ^a^ ± 55.4	139.5 ^a^ ± 45.3	139.5 ^b^ ± 39.2
55–64	2783 ^b^ ± 987	84.8 ^c^ ± 16.5	363.9 ^b^ ± 58.5	147.3 ^b^ ± 47.2	133.0 ^c^ ± 39.3
65–74	2791 ^b^ ± 1027	83.5 ^c^ ± 16.6	370.7 ^c^ ± 58.8	152.3 ^c^ ± 46.6	133.0 ^c^ ± 40.0
**Marital status**					
Married	2964 ^a^ ± 1010	86.9 ^a^ ± 15.8	359.5 ^a^ ± 55.5	139.3 ^a^ ± 44.5	139.3 ^a^ ± 39.2
Unmarried	2741 ^b^ ± 997	85.9 ^b^ ± 16.1	363.6 ^b^ ± 57.8	150.1 ^b^ ± 47.3	134.4 ^b^ ± 39.6
**BMI classification**					
Thinness	2975 ^ab^ ± 959	82.9 ^a^ ± 14.6	379.6 ^a^ ± 50.7	137.3 ^ab^ ± 45.5	157.1 ^a^ ± 39.2
Eutrophic	2817 ^a^ ± 972	85.7 ^a^ ± 15.5	365.6 ^b^ ± 54.0	145.7 ^a^ ± 44.5	139.3 ^b^ ± 40.0
Overweight	2924 ^b^ ± 1024	87.2 ^b^ ± 16.1	357.0 ^c^ ± 57.4	141.1 ^b^ ± 46.5	135.4 ^c^ ± 38.7
Obesity	2987 ^b^ ± 1064	87.4 ^b^ ± 16.5	356.8 ^c^ ± 58.1	140.1 ^b^ ± 46.2	137.7 ^bc^ ± 38.9
**Family history of Diabetes**					
No	2881 ± 995	86.5 ± 15.8	360.9 ± 56.3	143.3 ± 45.6	137.4 ± 39.1
Yes	2905 ± 1039	86.8 ± 15.9	360.5 ± 55.8	142.1 ± 45.6	138.1 ± 39.7
**Hypertension**					
No	2865 ^a^ ± 994	87.3 ^a^ ± 15.8	360.5 ± 55.7	143.8 ^a^ ± 45.3	137.5 ± 39.5
Yes	2948 ^b^ ± 1047	85.0 ^b^ ± 16.1	361.5 ± 57.5	140.6 ^b^ ± 46.5	138.3 ± 39.0
**Education level**					
Middle School	3092 ^a^ ± 1103	80.7 ^a^ ± 15.7	382.8 ^a^ ± 55.4	141.6 ± 51.1	150.5 ^a^ ± 40.2
High School	3128 ^a^ ± 1090	83.7 ^b^ ± 15.5	373.4 ^b^ ± 54.0	143.6 ± 47.9	146.0 ^b^ ± 39.3
University	2744 ^b^ ± 926	88.9 ^c^ ± 15.7	351.2 ^c^ ± 55.3	142.6 ± 43.6	131.6 ^c^ ± 38.1
**Per Capita Income**					
Up to 1 MW ^2^	3149 ^a^ ± 1080	83.6 ^a^ ± 15.4	373.4 ^a^ ± 53.9	140.1 ^a^ ± 47.7	149.0 ^a^ ± 39.5
From 1 to 2 MW	2917 ^b^ ± 992	86.4 ^b^ ± 15.7	362.9 ^b^ ± 55.2	143.7 ^b^ ± 45.4	139.5 ^b^ ± 38.9
More than 2 MW	2637 ^c^ ± 893	89.5 ^c^ ± 16.0	347.5 ^c^ ± 56.4	144.3 ^b^ ± 43.8	126.0 ^c^ ± 36.4
**Alcohol Consumption**					
No	2975 ^a^ ± 1056	85.5 ^a^ ± 16.2	375.8 ^a^ ± 55.7	151.6 ^a^ ± 46.4	145.3 ^a^ ± 40.4
Yes	2884 ^b^ ± 999	87.4 ^b^ ± 15.6	354.0 ^b^ ± 54.8	138.4 ^b^ ± 44.2	135.0 ^b^ ± 38.7
**Tobacco Consumption**					
No	2927 ± 1014	86.9 ^a^ ± 16.0	356.7 ± 57.0	138.7 ^a^ ± 42.9	136.5 ^a^ ± 39.1
Yes	2948 ± 1063	88.2 ^b^ ± 16.4	353.3 ± 57.2	132.1 ^b^ ± 48.6	139.2 ^b^ ± 40.2
**Leisure Physical Activity**					
Weak	2902 ± 1025	86.7 ^a^ ± 15.9	361.3 ^a^ ± 56.1	141.8 ^a^ ± 46.3	139.8 ^a^ ± 39.3
Moderate	2844 ± 961	85.1 ^b^ ± 16.0	362.3 ^a^ ± 56.5	146.6 ^b^ ± 44.1	132.4 ^b^ ± 39.0
Strong	2880 ± 981	87.0 ^a^ ± 15.8	353.7 ^b^ ± 57.7	145.6 ^ab^ ± 42.7	126.2 ^c^ ± 37.8

^1^ Data presented in Mean ± SD (standard deviation)—CHO: Carbohydrate. Student *t*-test for variables with two categories and ANOVA plus Tukey test for variables with more than two categories. Equal letters indicate no significant difference. Different letters indicate statistically significant differences. Differences expressed when *p* < 0.05 (analysis without the letter did not show statistical differences between the evaluated categories). ^2^ MW: Minimal Wage.

**Table 3:** Association between sociodemographic and lifestyle variables and nutrient intake (protein: animal and vegetal; fiber: soluble and insoluble). ELSA-Brazil. 2008–2010 ^1^.

	PTN (g)	Animal PTN (g)	Vegetal PTN (g)	Total Fiber (g)	Soluble Fiber (g)	Insoluble Fiber (g)
	Mean ± SD	Mean ± SD	Mean ± SD	Mean ± SD	Mean ± SD	Mean ± SD
**Gender**						
Male	123.6 ^a^ ± 24.4	80.7 ^a^ ± 29.1	42.9 ^a^ ± 9.8	34.7 ^a^ ± 11.0	13.9 ^a^ ± 11.3	25.8 ± 8.7
Female	127.9 ^b^ ± 25.7	86.6 ^b^ ± 30.1	41.6 ^b^ ± 9.5	35.9 ^b^ ± 11.2	18.8 ^b^ ± 13.7	26.1 ± 8.5
**Ethnicity**						
Black	123.4 ^a^ ± 25.4	81.1 ^a^ ± 29.8	42.3 ^ab^ ± 9.8	35.7 ^abc^ ± 11.2	14.4 ^a^ ± 11.9	26.0 ^ab^ ± 8.8
Brown	124.6 ^a^ ± 25.9	82.1 ^a^ ± 30.3	42.5 ^ab^ ± 9.8	35.1 ^a^ ± 10.5	15.5 ^a^ ± 12.5	25.8 ^a^ ± 8.5
White	127.5 ^b^ ± 24.7	85.8 ^b^ ± 29.3	42.0 ^a^ ± 9.5	35.3 ^ab^ ± 11.2	17.6 ^b^ ± 13.2	25.9 ^a^ ± 8.5
Yellow/Indigenous	122.4 ^a^ ± 25.4	78.9 ^a^ ± 30.0	43.6 ^b^ ± 9.6	37.3 ^c^ ± 12.1	15.6 ^a^ ± 12.7	27.3 ^b^ ± 9.2
**Age (years)**						
35–44	124.8 ^a^ ± 23.4	83.0 ^a^ ± 28.4	42.0 ^a^ ± 9.8	33.1 ^a^ ± 10.7	15.6 ^a^ ± 11.5	24.5 ^a^ ± 8.4
45–54	125.8 ^ab^ ± 24.8	83.8 ^a^ ± 29.5	42.1 ^a^ ± 9.6	34.6 ^b^ ± 10.6	15.8 ^a^ ± 12.7	25.5 ^b^ ± 8.3
55–64	127.0 ^b^ ± 26.3	84.7 **^a^** ± 30.7	42.4 ^a^ ± 9.5	37.1 ^c^ ± 11.2	17.3 ^b^ ± 13.5	27.1 ^c^ ± 8.6
65–74	125.5 ^ab^ ± 27.0	83.4 ^a^ ± 31.1	42.3 ^a^ ± 10.1	38.4 ^d^ ± 12.2	18.7 ^c^ ± 13.8	27.7 ^c^ ± 9.3
**Marital status**						
Married	125.5 ^a^ ± 24.3	83.2 ^a^ ± 29.1	42.3 ^a^ ± 9.6	35.2 ^a^ ± 11.0	15.8 ^a^ ± 12.2	25.9 ± 8.6
Unmarried	126.7 ^b^ ± 26.8	85.0 ^b^ ± 31.1	41.9 ^b^ ± 9.7	35.7 ^b^ ± 11.2	18.0 ^b^ ± 14.0	26.0 ± 8.6
**BMI classification**						
Thinness	116.9 ^a^ ± 24.4	70.0 ^a^ ± 29.1	46.8 ^a^ ± 11.4	36.9 ^ab^ ± 11.2	15.1 ^ab^ ± 11.9	27.8 ^a^ ± 9.4
Eutrophy	124.8 ^b^ ± 24.3	81.8 ^b^ ± 29.0	43.1 ^b^ ± 9.7	36.1 ^a^ ± 11.2	17.1 ^a^ ± 13.3	26.6 ^a^ ± 8.7
Overweight	126.7 ^c^ ± 25.8	85.3 ^c^ ± 30.2	41.5 ^c^ ± 9.4	34.9 ^b^ ± 10.9	16.3 ^b^ ± 12.6	25.6 ^b^ ± 8.3
Obesity	127.3 ^c^ ± 25.5	86.2 ^c^ ± 30.1	41.3 ^c^ ± 9.6	34.4 ^b^ ± 11.1	15.7 ^b^ ± 12.4	25.1 ^b^ ± 8.5
**Family history of Diabetes**						
No	125.6 ± 25.0	83.6 ± 29.6	42.1 ± 9.7	35.2 ± 11.2	16.7 ± 12.9	25.9 ± 8.6
Yes	126.6 ± 25.5	84.4 ± 29.8	42.3 ± 9.5	35.6 ± 10.9	16.3 ± 12.7	26.1 ± 8.4
**Hypertension**						
No	125.6 ± 24.8	83.5 ± 29.4	42.3 ± 9.6	35.3 ± 11.0	16.7 ^a^ ± 12.8	25.9 ± 8.5
Yes	126.4 ± 25.8	84.5 ± 30.4	42.0 ± 9.7	35.5 ± 11.2	16.0 ^b^ ± 13.0	26.0 ± 8.8
**Education level**						
Middle School	117.4 ^a^ ± 25.5	74.0 ^a^ ± 29.8	43.3 ^a^ ± 10.0	37.1 ^a^ ± 11.0	15.4 ^a^ ± 12.8	27.3 ^a^ ± 8.7
High School	121.4 ^b^ ± 24.3	78.2 ^b^ ± 28.5	43.2 ^a^ ± 9.8	35.9 ^b^ ± 11.3	15.2 ^a^ ± 12.1	26.5 ^b^ ± 8.8
University	129.4 ^c^ ± 24.9	88.1 ^c^ ± 29.5	41.6 ^b^ ± 9.5	34.8 ^c^ ± 11.0	17.3 ^b^ ± 13.2	25.5 ^c^ ± 8.4
**Per Capita Income**						
Up to 1 MW ^2^	120.5 ^a^ ± 23.8	77.2 ^a^ ± 28.0	43.2 ^a^ ± 9.7	35.2 ± 10.5	15.0 ^a^ ± 12.3	26.0 ± 8.4
From 1 to 2 MW	125.5 ^b^ ± 24.7	83.3 ^b^ ± 29.2	42.4 ^b^ ± 9.7	35.5 ± 11.4	16.8 ^b^ ± 13.0	26.0 ± 8.7
More than 2 MW	131.1 ^c^ ± 25.7	90.2 ^c^ ± 30.3	41.2 ^c^ ± 9.5	35.4 ± 11.4	17.6 ^c^ ± 13.1	25.8 ± 8.7
**Alcohol**						
No	124.1 ^a^ ± 25.7	81.1 ^a^ ± 30.5	43.1 ^a^ ± 10.3	36.2 ^a^ ± 11.7	17.5 ^a^ ± 13.1	26.6 ^a^ ± 9.1
Yes	126.6 ^b^ ± 24.8	84.8 ^b^ ± 29.4	41.8 ^b^ ± 9.4	34.9 ^b^ ± 10.9	15.9 ^b^ ± 12.6	25.6 ^b^ ± 8.4
**Tobacco**						
No	126.5 ^a^ ± 25.8	84.5 ^a^ ± 30.4	42.1 ^a^ ± 9.5	35.8 ^a^ ± 11.2	15.8 ^a^ ± 12.6	26.2 ^a^ ± 8.6
Yes	121.9 ^b^ ± 24.5	80.4 ^b^ ± 28.8	41.5 ^b^ ± 9.7	33.6 ^b^ ± 10.6	13.9 ^b^ ± 12.6	24.9 ^b^ ± 8.4
**Leisure Physical Activity**						
Weak	124.9 ^a^ ± 24.8	82.9 ^a^ ± 29.4	42.1 ^a^ ± 9.6	34.7 ^a^ ± 10.8	16.2 ^a^ ± 12.8	25.4 ^a^ ± 8.4
Moderate	128.2 ^b^ ± 25.2	85.5 ^b^ ± 29.8	42.8 ^b^ ± 9.5	38.0 ^b^ ± 11.6	17.4 ^b^ ± 13.4	27.8 ^b^ ± 8.8
Strong	132.3 ^c^ ± 27.0	89.9 ^c^ ± 32.4	42.6 ^ab^ ± 9.9	37.5 ^b^ ± 11.9	17.6 ^b^ ± 12.8	27.5 ^b^ ± 9.1

^1^ Data presented in Mean ± SD (standard deviation)—PTN: Protein. Student *t*-test for variables with two categories and ANOVA plus Tukey test for variables with more than two categories. Equal letters indicate no significant difference. Different letters indicate statistically significant differences. Differences expressed when *p* < 0.05 (analysis without the letter did not show statistical differences between the evaluated categories). ^2^ MW: Minimal Wage.

**Table 4:** Characterization of the daily consumption of energy and macronutrients by ELSA-Brasil participants according to the use of nonnutritive sweeteners (2008–2010).

Characteristics		Regular Use of NNS ^1^			
	Total (n = 9226)	No (n = 6852)	Yes (n = 2374)	∆ (Yes-No)	*p*-Value ^2^
	Mean ± SD	Mean ± SD	Mean ± SD		
Kcal	2891 ± 1012	2949 ± 1022	2725 ± 96	−2245	<0.001
Fats (g)	86.6 ± 15.9	85.7 ± 15.5	89.0 ± 16.8	3.3	<0.001
Carbohydrates (g)	360.8 ± 56.3	365.9 ± 54.8	346.2 ± 58	−19.7	<0.001
Simple Carbohydrates (g)	142.8 ± 45.7	145.2 ± 46.1	135.7 ± 43.8	−9.5	<0.001
Complex Carbohydrates (g)	137.7 ± 39.4	139.8 ± 39.6	131.6 ± 38.1	−8.2	<0.001
Protein (g)	125.9 ± 25.2	122.6 ± 24.0	135.3 ± 26.0	12.7	<0.001
Animal Protein (g)	83.8 ± 29.7	80.3 ± 28.5	94.1 ± 30.8	13.8	<0.001
Vegetal Protein (g)	42.2 ± 9.7	42.4 ± 9.7	41.5 ± 9.3	−0.9	<0.001
Total Fiber (g)	35.4 ± 11.1	35.2 ± 11.2	35.7 ± 10.9	0.5	0.09
Soluble Fiber (g)	16.5 ± 12.9	15.7 ± 12.4	18.8 ± 14.0	3.1	<0.001
Insoluble Fiber (g)	25.9 ± 8.6	25.9 ± 8.7	26.0 ±8.3	0.1	0.7

^1^ NNS: Nonnutritive Sweeteners. ^2^ Student *t*-test.

**Table 5:** Association between the regular consumption of nonnutritive sweeteners (NNSs) and dietary intake among non-diabetic participants from the Brazilian Longitudinal Study of Adult Health—ELSA-Brasil. 2008–2010.

Variables	Unadjusted Model			Adjusted Model *		
	r^2^	β (95% CI)	*p*	r^2^	β (95% CI)	*p*
Kcal	0.009	−226.64 (−274.0; −179.3)	<0.001	0.140	−74.29 (−147.86; −0.71)	0.048
Fats (g)	0.002	−3.81 (−5.55; −2.07)	<0.001	0.084	−0.46 (−3.27; 2.35)	0.749
Carbohydrates (g)	0.022	−50.86 (−57.76; −43.97)	<0.001	0.159	−23.68 (−34.28; −13.09)	<0.001
Simple Carbohydrates (g)	0.008	−9.54 (−11.67; −7.42)	<0.001	0.115	−11.24 (−14.48; −8.00)	<0.001
Complex Carbohydrates (g)	0.008	−8.21 (−10.03; −6.38)	<0.001	0.123	−1.87 (−4.73; 0.99)	0.199
Protein (g)	0.007	3.10 (0.62; 5.59)	<0.001	0.071	7.38 (3.43; 11.34)	<0.001
Animal Protein (g)	0.056	7.63 (5.56.85; 9.69)	<0.001	0.043	8.56 (5.21; 11.92)	<0.001
Vegetal Protein (g)	0.107	−4.40 (−5.27; −3.54)	<0.001	0.145	−1.14 (−2.49; 0.20)	0.945
Soluble Fiber (g)	0.003	−2.22 (−3.02; −1.42)	<0.001	0.089	0.28 (0.66; 1.55)	0.666

* Model adjusted for variables: sex, race/color, age, marital status, body mass index (BMI), systemic arterial hypertension, schooling, per capita income, current alcohol consumption, current tobacco consumption, and leisure-time physical activity.

## Data Availability

Due to ethical restrictions approved by the ethics committee of each institution (Universidade Federal de Minas Gerais, Universidade de São Paulo, Universidade Federal do Espírito Santo, Universidade Federal do Rio Grande do Sul, Universidade Federal da Bahia e Fundação Oswaldo Cruz) and by the Publications Committee of ELSA-Brasil (publiELSA), the data used in this study can be made available for research proposals by a request to ELSA’s Datacenter (rb.sgrfu@asleacitsitatse) and to the ELSA’s Publications Committee. Additional information can be obtained from the ELSA Coordinator from the Research Center of Espírito Santo (jose.mill@gmail.com).
